# PIMS-TS, the New Paediatric Systemic Inflammatory Disease Related to Previous Exposure to SARS-CoV-2 Infection—“Rheumatic Fever” of the 21st Century?

**DOI:** 10.3390/ijms22094488

**Published:** 2021-04-26

**Authors:** Violetta Opoka-Winiarska, Ewelina Grywalska, Jacek Roliński

**Affiliations:** 1 Department of Paediatric Pulmonology and Rheumatology, Medical University of Lublin, Gębali 6, 20-093 Lublin, Poland; 2 Department of Clinical Immunology and Immunotherapy, Medical University of Lublin, Chodzki 4a Street, 20-093 Lublin, Poland; ewelina.grywalska@umlub.pl (E.G.); jacek.rolinski@umlub.pl (J.R.)

**Keywords:** COVID-19, paediatric inflammatory multisystemic syndrome (PIMS), rheumatic fever

## Abstract

Paediatric inflammatory multisystem syndrome temporally associated with *severe acute respiratory syndrome coronavirus 2* (*SARS-CoV-2*) (PIMS-TS) is a new systemic inflammatory disease that mainly affects children. Its course in many features resembles that of acute rheumatic fever (ARF). Therefore, it is interesting that the experiences with ARF can be used in the management of patients with PIMS-TS. The aim of the article is to analyse the current data on PIMS-TS in relation to ARF. PIMS-TS and ARF are associated with an abnormal immune response to specific pathogens (*SARS-CoV-2* and group *A streptococcus*, respectively). The main symptoms of both diseases are fever and cardiac involvement. Current therapy for PIMS-TS is based on anti-inflammatory treatment: intravenous immunoglobulin (first-line), intravenous glucocorticoids (second-line), or biological therapy (third-line; including interleukin [IL]-1 antagonists, IL-6 receptor blockers, and anti-tumour necrosis factor agents). Vaccination might be good prophylaxis, but the efficacy and safety of the vaccines against *SARS-CoV-2* have not yet been established in children. Interesting insights may be gained by considering PIMS-TS in light of what is known of ARF due to their similar courses, but there are still many unanswered questions surrounding this disease and its pathogenesis.

## 1. Introduction

Paediatric inflammatory multisystem syndrome temporally associated with *severe acute respiratory syndrome coronavirus 2* (*SARS-CoV-2*) (PIMS-TS) is a new systemic inflammatory acute onset disease that mainly affects children. The definitions and diagnosis criteria have been developed in 2020 by the UK Royal College of Paediatrics and Child Health [1], the US Center for Disease Control and Prevention (CDC) [2], and the World Health Organization (WHO) [3]. The name proposed for this disease by the CDC and WHO is slightly different: multi-system inflammatory syndrome in children (MIS-C). The disease usually occurs in children 3–6 weeks after an acute *SARS-CoV-2* infection (usually mild) or contact with an infected person [4].

The main symptom of PIMS-TS is fever, but its course may be similar to that of other autoinflammatory diseases, such as complete, incomplete, and atypical Kawasaki disease, toxic shock syndrome, sepsis, hemophagocytic syndrome [5] or systemic onset juvenile idiopathic arthritis. Nevertheless, none of these other diseases have been linked to one specific pathogen.

For the practitioner, the course of PIMS-TS also resembles that of acute rheumatic fever (ARF), also a paediatric inflammatory disease. The clinical description of ARF was first described in 1889 and first criteria for its diagnosis were established by Jones in 1944 [6]. The experience and knowledge of ARF is therefore much greater than the PIMS-TS, but it is not without questions.

ARF is considered a model of infection-induced autoimmune diseases [7]. This multiorgan acute inflammatory disorder is a delayed consequence of a throat infection with group *A streptococcus* (*GAS*). The latent period following pharyngitis usually lasts 2–3 weeks before the first symptoms of ARF appear. The main symptom of the disease, observed in more than 90% of patients, is fever. Other symptoms may include heart inflammation, arthritis, neurologic disorders (chorea), and skin involvement (subcutaneous nodules and marginal erythema). Heart valve disease can be chronic and progressive, eventually resulting in permanent valve damage and heart failure [8]. The classic triad of events consists of a *GAS* throat infection, a genetically susceptible host, and an abnormal host immune response [7].

PIMS-TS and ARF are undoubtedly different diseases, but they share some similarities [9], including certainty regarding the associated pathogen, the occurrence in childhood, the mild course of the infection preceding the disease, and the occurrence of symptoms after a few asymptomatic weeks after the infection (Table 1).

Therefore, it is interesting that the experiences with ARF can be used in the management of patients with PIMS-TS. Can our medical knowledge with ARF help in understanding PIMS-TS? The aim of this article is to analyse the current data on PIMS-TS in relation to ARF.

## 2. Relationship with Infection

The basic condition for the diagnosis of both PIMS-TS and ARF is confirmation of prior infection, *SARS-CoV-2* or *GAS*, respectively [2,3,10] (Table 1).

The criteria for PIMS-TS/MIS-C diagnosis according to the CDC and WHO require demonstration of association with *SARS-CoV-2* infection by exposure to coronavirus disease 2019 (COVID-19), positive serological test or prior RT- PCR or antigen test for SARS-CoV-2 [2,3]. Most patients have negative PCR test results for *SARS-CoV-2*, but 83–99% have positive serology [4]. In the largest published study on children with PIMS-TS (MIS-C) (570 cases), the association with *SARS-CoV-2* (by RT-PCR or serology) was confirmed in all the children who were tested (99.1%) [11].

Consistent with previous exposure to *SARS-CoV-2* and with observations of the serological response during convalescence, PIMS-TS patients had elevated levels of immunoglobulin G (IgG), with dominant IgG1, and low levels of IgM antibodies. However, compared with other convalescents, PIMS-TS patients showed lower IgM levels, and their IgA titres were higher and similar to the IgA levels observed in acute infection. The plasma of all PIMS-TS patients was also neutralizing, with a potency similar to that of convalescent adults [12].

The Jones Criteria, last revised in 2015, remain the basis for the diagnosis of ARF [10]. Evidence of preceding *GAS* infection is required for the diagnosis of ARF. This can be confirmed by an increased or rising titre of anti-streptolysin O (ASO) or other streptococcal antibodies (anti-deoxyribonuclease B [anti-DNase B]), an earlier positive throat culture for *GAS*, or a positive rapid *GAS* carbohydrate antigen test in a child with clinical presentation of streptococcal pharyngitis [10,11].

The *SARS-CoV-2* infection in children is not specific, and it is difficult to distinguish from infections from other viruses. European data show (as of 10 August 2020), that children aged <10 years accounted for 1.9% of total patients and children aged 10–19 constituted 3.7% of total patients [13], although the true incidence may be higher.

The diagnosis of mild or asymptomatic infections is complicated by the decrease in IgG against *SARS-CoV-2* protein observed up to 3 months after the onset of symptoms in non-severe or asymptomatic patients. Furthermore, a weaker immune response to the *SARS-CoV-2* infection was demonstrated in these patients compared to the seriously ill [14].

The percentage of children suffering from PIMS-TS after COVID-19 has not yet been established, but it remains a rare disease. The median age of patients with PIMS-TS is 6.6–10 years (range 3.7–16.6 years), and 43–74% of them are male. Regarding ethnicity, 40.5% of patients ae Hispanic, 33.1–73.9% ae black no-Hispanic, 13.2–29% are white no-Hispanic, and 2.8–10% are Asian [4].

*GAS* is the most common cause of bacterial pharyngitis in children and adolescents. Among children of all ages who present with a sore throat, the prevalence of *GAS* is estimated at 37% [15]. Depending on the genetic predisposition of the patients and the bacterial virulence, about 0.3–3% of patients with *GAS* pharyngitis develop ARF [8].

Throat cultures are negative in about 75% of patients by the time manifestations of ARF are observed. Following *GAS* pharyngitis, the antibody (ASO and anti-DNase B) response peaks at 4–5 weeks, which usually corresponds to the second or third week of ARF. Antibody titres decline in the following months, usually up to the 6th month for ASO and up to the 6th–9th month for anti-DNase B. About 90% of patients with documented ARF have positive test results if two antigens are evaluated [16].

The incidence of ARF is highest among children aged 5–14 years, similar to that of PIMS-TS, although it can also occur in adults. Children younger than 5 years rarely develop ARF. The disease is not linked to gender, but rheumatic heart disease (RHD) is more common in females [7,8].

Questions awaiting answer

□ How often is PIMS-TS a complication of COVID-19 in children?
□ What age-related features of the immune system predispose to excessive activation of inflammation after *SARS-CoV-2* infection?
□ What is the role of anti-*SARS-CoV-2* antibodies in the pathogenesis of the disease?

## 3. Clinical Symptoms

No single laboratory test or clinical feature is diagnostic of PIMS-TS or ARF. The main symptoms of both diseases are fever and cardiac involvement. Both diseases can develop symptoms from other organs. Elevated markers of systemic inflammation in laboratory tests are common. The criteria for diagnosis also have common features [1,2,3,10] (Table 1).

PIMS-TS symptoms are diverse and non-specific. The main symptom of the disease and the basic diagnostic criterion is fever [1,2,3].

Cardiovascular symptoms are the hallmark of PIMS-TS patients. They occur in about 86.5% of patients and are associated with the life-threatening course of the disease. Cardiovascular symptoms can have different manifestations involving coronary artery dilatation or aneurysms (18.6%), myocarditis (22.8%), left ventricular dysfunction (40.6%), pericardial effusion (23.9%), mitral regurgitation (25.5%), hypotension (49.5%), and shock (35.4%).

Other symptoms involve the gastrointestinal system (abdominal pain, vomiting, diarrhoea), skin and mucous membranes (rash, conjunctival injections, cheilitis, and stomatitis), respiratory system (cough, chest pain, pneumonia, pleural infusion, chest pain, respiratory failure), neurological system (headache, meningism), renal system (acute kidney injury), lymphadenopathy, and others [4].

Fever is seen in more than 90% of patients with ARF and belongs to the Jones criteria for the diagnosis of disease [10]. ARF can cause pancarditis (also one of the Jones criteria), which involves the pericardium, myocardium, and endocardium, including valvular inflammation. Carditis is diagnosed clinically in 50–70% of cases, subclinical carditis in an additional 12–21%. Taken together they affect up to 90% of patients. Pericarditis resolves without sequelae. Endocardial involvement is main manifestation of ARF carditis. It may present as valvulitis of the mitral valve or less frequently, of the aortic valve and cause their regurgitation. Clinical manifestations of carditis may be palpitations, dyspnoea or left heart failure [7]. RHD is the most severe sequelae of ARF. It usually occurs 10–20 years after the onset of the disease and is the most common cause of acquired valvular disease. In turn, the most commonly pathology in RHD is mitral stenosis, caused by severe scarring and calcification of the mitral valve. Valvular occur in about 50% of patients with carditis at initial presentation [7,10,15].

Chorea is a neurologic disorder consisting of involuntary, purposeless movements of the extremities and trunk, muscular weakness, and emotional disturbances, without sensory losses or involvement of the pyramidal tract. Chorea can present up to 3 months after an ARF episode or can occur as an isolated manifestation. [8,10,15]

Arthritis due to ARF consists of migratory polyarthritis involving the large joints (knees, ankles, elbows, and wrists). The most prominent feature of arthritis is transient joint pain.

Skin manifestations characteristic of ARF include erythema marginatum (an evanescent, pink, non-pruritic rash that occurs mainly on the trunk and proximal extremities) and subcutaneous nodules (small, firm, painless lesions usually located over a bony surface or prominence or near tendons) [8,10,15]. Abdominal pain, a rapid sleeping pulse rate, tachycardia out of proportion to fever, malaise, epistaxis, and precordial pain may also occur in patients with ARF [10].

Patients with PIMS-TS have characteristic laboratory features, including elevated inflammatory markers, such as C-reactive protein (CRP), erythrocyte sedimentation rate (ESR), and ferritin. Evidence of inflammation is part of the criterion used for disease diagnosis. Other common findings include lymphopenia, neutrophilia, and thrombocytopenia. Inflammatory cytokine levels are often elevated, including interleukin (IL)-6, tumour necrosis factor α (TNF-α), and IL-10. Patients with PIMS-TS have elevated D-dimer values which may be related to increased incidence of thromboembolic events [1,2,3]. Other markers of inflammation characteristic of this disease are frequently elevated, including procalcitonin (PCT) with no bacterial infection. [4].

The clinical features of ARF are accompanied by evidence of inflammation. Elevated CRP or ESR are minor Jones criteria for ARF [6,9]. They are useful for monitoring inflammation. Other symptoms observed in patients with ARF include mild anaemia related to chronic inflammation and leucocytosis [10,15].

Questions awaiting answer

□ Can PIMS-TS symptoms recur?
□ Will subsequent *SARS-CoV-2* infections in a child with a history of PIMS-TS cause recurrence of symptoms?
□ What are the late consequences of PIMS-TS?
□ Can PIMS-TS cause chronic changes in the cardiovascular system?

## 4. Pathogenesis

Infectious agents are considered an important environmental factor potentially triggering autoimmune and autoinflammatory diseases, mainly through molecular mimicry [17]. However, it is usually not a single infection. PIMS-TS and ARF are caused by an abnormal immune system response to one specific pathogen (*SARS-CoV-2* and *GAS*, respectively) in people who are genetically or otherwise predisposed.

### 4.1. Genetic Factors

Whether a genetic predisposition influences the development of PIMS-TS is yet to be established, but it has been shown that Black or Hispanic ethnicity may be a risk factor [12]. Several susceptibility genes: *ITPKC, CASP3, CD40, BLK* and *FCGR2A*, and *HLA* class II alleles have been associated with Kawasaki disease. Combinations of gene variants may be genetic biomarkers for identifying the risk of coronary artery lesions and refractory disease [18]. Perhaps variants of these genes are also associated with the risk of PIMS-TS, although a lower incidence of the disease is observed in Asian children compared to children of other ethnicities [4,12].

Several genes have been associated with the incidence of ARF, both alleles encoding proteins involved in innate and acquired immune responses: *TLR2, FCN2, MASP2, MBL2, MIF*, and *FCGR2A* (innate immunity), *HLA* class II alleles and IGHV4-61*02 (adaptive immunity), *IL1RA,* *TNF-α, TGFB1, IL10*, and *CTLA4* (innate and adaptive immunity) [7].

Questions awaiting answer

□ What are the genetic risk factors in PIMS-TS?
□ Do certain variants of genes encoding proteins involved in primary and acquired immunity play a role in the pathogenesis of PIMS-TS?
□ Are the genetic factors associated with a variant form of PIMS-TS?
□ Could genetic testing be used in PIMS risk assessment?

### 4.2. The Role of Inflammatory Cytokines

According to current reports, the symptoms of PIMS-TS and ARF occur as a result of both humoral and cellular immune responses against proteins of self-antigens.

Cytokine profiling in PIMS-TS patients indicates myeloid cell chemotaxis and inflammation, although reports on the subject are missing. An assessment of cytokines in patients with PIMS-TS by Gruber et al. [12] showed an increased release of the pro-inflammatory cytokines IL-6, IL-18, and IL-17A and the chemokines CCL19, CXCL10, and CDCP1, which regulate the recruitment and modulation of natural killer (NK) cells and T lymphocytes. They also observed an increased release of CCL3, CCL4, and CDCP1, which mediate neutrophil and monocyte chemotaxis, and secretion of EN-RAGE and CSF-1, which affect neutrophil and monocyte differentiation and activity. A significant increase in soluble programmed death-ligand 1 indicates exhaustion and immunosuppression, possibly reflecting a compensatory inflammatory response. Importantly, almost all increases in PIMS-TS cytokines resolved to healthy levels after discharge from hospital [12]. In a study by Diorio et al. [19] the cytokine profile associated with PIMS-TS showed elevated levels of IL-6, IL-8, IL-10, and TNF-α. Other observations published by Rolando-Cruz et al. [20] showed markedly elevated levels of IL-6 and IL-8 in in patient with PIMS-TS. In contrast, TNF-α was only minimally elevated and no patients had elevated levels of IL-1. An analysis of serum cytokines by Lee et al. [21] showed elevated IL-6, IL-10, and soluble IL-2 receptor levels.

The IL-6 and IL-1 assays are useful as inflammatory markers in the diagnosis and assessment of disease activity. Blocking pro-inflammatory cytokines (IL-6, IL-1, TNF-α) is one of the therapies recommended for PIMS-TS [5,22].

As in PIMS-TS, pro-inflammatory cytokines play a key role in the pathogenesis of ARF. As is currently known, local tissue damage in ARF is mediated mainly by T1 helper cells responses, leading to the release of inflammatory cytokines such as interferon γ (INF-γ) and TNF-α, and reduced release of IL-4 and IL-10 [7]. According to Guilherme at al. [7,23] in patients with RHD resulting from ARF, local inflammation triggered by autoimmune reaction could be intensify by TNF-α and IFN-γ produced by mononuclear cells.

Autoantibodies bound to the cell surface induce release of vascular cell adhesion molecule 1 (VCAM-1) which causes adherence and infiltration of activated T and B lymphocytes [24]. Th17 cells and increased release of IL-17 are also involved in pathogenesis ARF and RHD [25].

Questions awaiting answer

□ Why and by what mechanism does the virus cause an inflammatory response in the post-infection period?
□ Can other molecules involved in the pathogenesis of PIMS-TS be used as markers for the early diagnosis and assessment of disease activity?
□ Will the understanding of key cytokines and inflammation pathways lead to other targeted therapies?

### 4.3. Immune Cell Activation

Lymphocyte immunotyping in patients with PIMS-TS revealed changes in the number and frequency of some types of immune cells. Research by Gruber et al. [12] showed that in PIMS-TS patients the percentages of Tλδ and Tαβ lymphocytes were reduced relative to healthy controls, but the relative distribution of naive central memory cells was normal. B lymphocytes were present in normal numbers and frequencies and had the typical distribution of naive, memory, and plasma cells. Among innate cells, CD56^lo^ NK cell counts were reduced, while frequencies of non-classical monocytes and plasmacytoid dendritic cells were lower [12].

Similarly, in the study by Lee et al. [21], most patients with PIMS-TS exhibited reduced numbers of CD4+ and CD8+ T lymphocytes and NK cells, whereas B lymphocytes were reduced less consistently.

Comparing markers of immune function in PIMS-TS patients and healthy participants revealed an upregulated CD54 (ICAM-1) expression on neutrophils and CD16+ monocytes, indicative of antigen presenting cell activation and trans-endothelial migration. Also, neutrophils and CD16+ non-classical monocytes demonstrated elevated CD64 (FcRg1) expression in patients, a common finding in autoimmune and autoinflammatory diseases. CD64 can engage autoantibodies and immune complexes to trigger potent inflammation and tissue injury [12]. These cells lacked signs of active type I IFN signalling, including CD169 and STAT1 phosphorylation upregulation, suggesting other cytokines are driving this activation. Instead, some patients had augmented levels of phospho-STAT3 [12]. Together, these findings suggest that the active peripheral expansion of subsets of NK and T lymphocytes, but not B lymphocytes, and activation and chemotaxis of neutrophils and nonclassical monocytes likely contribute to the underlying disease pathogenesis.

The frequency changes in these types of cells resolve a few weeks after discharge in patients with PIMS-TS. These results suggest that the inflammatory innate immune response and autoreactivity secondary to *SARS-CoV-2* infection may be critical to the pathogenesis of PIMS-TS.

Nevertheless, this description is based on a single study [12] and requires confirmation in subsequent studies.

*GAS* throat infection triggers the innate and adaptive immune responses by activating neutrophils, monocytes and macrophages, and B and T lymphocytes. In ARF autoreactive T lymphocytes can react with self-antigens. Lymphocytes T infiltration is connected with Th1 response. Patients with RHD have increased numbers of Th17 cells [6]. Guilherme et al. [23] showed that, in RHD patients, heart lesions contain T-cell clones that recognize heart proteins and streptococcal M peptides. The heart-infiltrating T lymphocytes characterized by IFN-γ and TNF-α and IL-10-positive cells were consistently predominant, that suggests that Th1 response could mediate RHD [19].

Questions awaiting answer

□ Are the innate and acquired immune disorders in PIMS-TS only secondary to the infection, or are they also primary?

### 4.4. Autoantibodies Target Organ Systems Central to PIMS-TS and ARF Pathology

The delayed onset after *SARS-CoV-2* infection and the resolution of the disease with intravenous immunoglobulin (IVIG) suggest a pathological process involving adaptive immunity. One of the hypotheses regarding the pathogenesis of PIMS-TS assumes that infection with *SARS-CoV-2* leads to a secondary autoreactive humoral response. The presence of autoantibodies has been demonstrated in patients with PIMS-TS, both against autoantigens associated with autoimmune diseases and antigens recognizing endothelial, gastrointestinal tract, and immune cells [12].

Gruber et al. [12] found nearly 300 potentially important autoantibodies in PIMS-TS patients, including anti-La and anti-Jo-1 antibodies occurring in classic rheumatic diseases. According to the authors, the results may indicate a link between the pathophysiology of PIMS and classical autoimmune diseases. More importantly, the most autoreactive antibodies in PIMS-TS patients are directed against antigens of organs important in PIMS-TS pathology such as the endothelium of the heart (P2RX4, ECE1 and MMP14), the gastrointestinal tract (MUC15, TSPAN13 and SH3BP1) and immune cell receptors (CD244, IL-1A, IFNGR2, IL-6R and LAMP1) [11].

Inflammation in ARF is also the result of a cross-immune response resulting from sequence similarity between *GAS* antigens and self-peptides, defined as molecular mimicry. In genetically predisposed individuals, immune response primarily to *GAS* antigens activates humoral and cellular immune pathways, leading to the production of antibodies that cross-react with human proteins (e.g., cardiac tissues). This leads to inflammation and immune-related injury [8].

The further course of disease and risk of PIMS-TS recurrence are unknown. The presence of autoantibodies in PIMS-TS patients is alarming. Gruber et al. postulate that autoantibodies can cause the formation of immune complexes or trigger the activity of immune cells on host tissues and organs. They may also arise as a result of direct cross-reactivity between *SARS-CoV-2* and autoantigens. While the inflammation appears to be transient, these autoantibodies also raise concerns about relapse or a predisposition to other disorders with autoimmune characteristics [12].

The pathogenesis of ARF has not yet been fully elucidated. Nevertheless, it is considered a model of infection-induced autoimmune diseases because the infectious agent is proven.

The autoimmune background of the disease is supported by the presence of anti-*GAS* antibodies in patients with rheumatic valvular disease. Antibodies and complement deposits have been detected in the heart of patients with RHD [7]. The streptococcal M protein is structurally and immunologically similar to cardiac proteins and acts as an antigen mimic, also initiating a cellular immune response that has been observed in animal studies [26]. Molecular mimicry is considered a key element of pathogenesis, as it occurs between the M protein of streptococcus and several proteins of the heart (tropomyosin, keratin, cardiac myosin, laminin, vimentin). The complement cascade appears to be activated by the lectin pathway [9]. Molecular mimicry also underlies the cell-mediated immune inflammation that occurs in ARF. T lymphocytes react with myosin and valve-derived proteins and release inflammatory cytokines. In addition, 40% of patients with RHD had antibodies against antigens of endothelial cells. Through molecular mimicry, these antibodies can contribute to endothelial damage and exposure of the basement membrane and extracellular matrix antigens [9].

Valve damage in ARF is the result of both humoral and cellular immune responses against valve proteins. The adherence and infiltration of activated peripheral T and B lymphocytes is regulated by interactions with adhesion molecules, such as vascular cell adhesion molecule 1 (VCAM-1) [7,8]. VCAM-1 is expressed in the valvular endothelium of rheumatic valves, and the CD4+ and CD8+ T cells present on the surface of the valve adhere to the endothelium and infiltrate the valves [8,27]. Local tissue injury is mediated mainly by the response of T-helper lymphocytes, leading to the production of inflammatory cytokines such as INF-γ and TNF-α and reduced concentrations of IL-4 and IL-10 [8].

Similarly, antibodies against *GAS* N-acetyl-beta-D-glucosamine cross-react with neuronal cells in the basal ganglia and cause the release of excess dopamine, which leads to chorea. The accumulation of immune complexes may result in migrating arthritis in ARF [8].

Molecular mimicry is also one of the hypotheses for the pathogenesis of PIMS-TS. *SARS-CoV-2* may act as a direct trigger for autoimmune and/or autoinflammatory conditions through molecular mimicry, for example against ACE receptors in heart tissue [9]. Another hypothesis is that inflammation and a dysregulated immune response following *SARS-CoV-2* infection may lead other environmental injuries to induce generalized inflammation and symptoms in predisposed individuals [17]. Another hypothesis is that *SARS-CoV-2* may present superantigenic fragments similar to those of the staphylococcal enterotoxins B that can trigger an inflammatory response [9].

Questions awaiting answer

□ What are the molecular mechanisms that trigger autoimmune and/or autoinflammatory responses following *SARS-CoV-2* infection?
□ Why do some patients develop autoantibodies after *SARS-CoV-2* infection?
□ What role do antibodies against *SARS-CoV-2* antigens play in the pathogenesis of PIMS-TS?
□ Do antibodies modulate immune cell activity or immune complex formation?
□ Can the presence of autoantibodies herald the development of an autoimmune disease?
□ Does PIMS-TS predispose to autoimmune disease?

## 5. Treatment

The goal of PIMS-TS treatment is to stabilize life-threatening manifestations and prevent long-term sequelae, including coronary artery aneurysms, myocardial fibrosis/scarring, and fixed cardiac conduction abnormalities [5,22].

According to current recommendations [5,22], children with PIMS-TS who are *SARS-CoV-2* positive on RT-PCR or antigen testing might be considered for antiviral therapy. Remdesivir is the first-choice antiviral therapy for *SARS-CoV-2*. Low-dose aspirin should be continued for a minimum of 6 weeks in all patients with PIMS-TS.

First-line anti-inflammatory therapy for all children with PIMS-TS is IVIG. Second-line therapy is intravenous methylprednisolone, and it is considered for children who remain unwell 24 h after infusion of IVIG, particularly if they have ongoing fever. Biological therapy (IL-1 antagonists, IL-6 receptor blockers, or anti-TNF agents) should be considered as a third-line option [5]. Patients with PIMS-TS with prothrombotic risk factors might benefit from anticoagulant prophylaxis. In Del Borello et al. study prophylactic anticoagulation therapy with enoxaparin was introduced in patients with PIMS-TS [28].

Treatment of ARF consists of anti-inflammatory therapy, antibiotic therapy, and heart failure management. Anti-inflammatory treatment consists of aspirin and corticosteroids. Treatment with aspirin is usually required for 1–4 weeks but can be given for up to 12 weeks. The efficacy of other anti-inflammatory drugs, including corticosteroids, in the setting of active rheumatic carditis remains unclear. No reduction in the risk of heart valve lesions was observed with corticosteroids or IVIG, although corticosteroids may be used in cases of severe carditis. Anti-inflammatory therapy should be continued until all symptoms have resolved. Normalization of inflammatory markers, such as ESR and CRP, is considered a biomarker of resolution [16].

## 6. Vaccination as Prophylaxis

Vaccines are potentially the most effective method of reducing disease. Despite the relatively short duration of the COVID-19 epidemic, in less than a year the global effort of scientists has resulted in the development and widespread use of two vaccines to prevent severe COVID-19. Both vaccines consist of modified RNA that encodes a version of the *SARS-CoV-2* spike protein containing mutations that lock the protein into a conformation that can induce neutralizing antibody responses. Studies have shown that they induce both humoral and cellular immunity [29,30]. Many other vaccines are still under clinical trials.

To date, studies of COVID-19 vaccines have been conducted in adults. For this reason, the efficacy and safety of COVID-19 vaccination in children is unknown. A safe and effective dose for children has not been established, and the length of time the vaccine will remain effective is unknown. Currently, clinical trials of the vaccine are being conducted among children and adolescents.

Prevention of ARF (primary prevention) relies on the correct diagnosis and antibiotic treatment of *GAS* pharyngitis. After the first ARF episode, patients are at high risk of recurrence, with progression to RHD. The most effective method to limit progression to RHD is the prevention of recurrent *GAS* pharyngitis with long-term antibiotics (secondary prevention) [15].

Vaccination to prevent *GAS* pharyngitis and invasive infection is still in the pre-clinical development phase [8] despite many years of studies. The vaccine could replace the current antibiotic prophylaxis for ARF.

Questions awaiting answer

□ Will vaccines be safe in children considering the different immune response to *SARS-CoV-2* infection?
□ Would it be possible that immune response to COVID-19 vaccination could trigger PIMS-TS?
□ Are booster doses needed to maintain immunity?
□ Does the vaccine prevent asymptomatic disease and limit transmission?
□ Will vaccinating children prevent PIMS-TS?

## 7. Conclusions

We have shown similarities between the pathogenesis, symptoms and course of both diseases. PIMS-TS is a new and rare systemic inflammatory disease that mainly affects children. It resembles ARF in that both are caused by an abnormal immune response to specific pathogens. Like in ARF, the main symptoms of PIMS-TS are fever and cardiac involvement. Elevated inflammatory markers are characteristic of PIMS-TS, and like in ARF, there is immune cell involvement. Adaptive immunity is likely involved in the pathogenesis of PIMS-TS. *SARS-CoV-2* infection may lead to a secondary autoreactive humoral response. Unlike in other autoimmune diseases, the autoantibodies in PIMS-TS patients target organ systems central to the pathology this disease. Like in ARF, molecular mimicry may also be involved.

Both diseases require anti-inflammatory therapy. IVIG is the first-line anti-inflammatory therapy in PIMS-TS; intravenous methylprednisolone is second-line therapy; and biological therapy, including IL-1 antagonists, IL-6 receptor blockers, and anti-TNF agents, is third-line therapy. Vaccination might be good prophylaxis, but the efficacy and safety of the COVID-19 vaccines have not yet been established in children. There are still many unanswered questions surrounding this disease and its pathogenesis. We have posed questions resulting from the above similarities, and the answer to them will prove very important in terms of the effective management of PIMS-TS.

## Figures and Tables

**Table 1:** Comparison of PIMS-TS and ARF [1,2,3,4,5,6,7].

Characteristic Feature		PIMS-TS	ARF
Definition	confirmed pathogen exposure *	*SARS-CoV-2*	*GAS*
		RT-PCR	pharyngeal swab
		antigen test	antigen test
		serology positive 100% IgG: 2–12 week	90% ASO: 4–24 week anti-DNase B: 4–36 week
	age of onset	childhood *	childhood, rarely in young adults
	fever *	yes (100%)	yes (>90%)
	symptoms of the cardiovascular system *	coronary artery dilatation or aneurysms, myocarditis, left ventricular dysfunction, pericardial effusion, mitral regurgitation, hypotension, shock	pancarditis: pericarditis, myocarditis, endocarditis, including valvular inflammation, heart failure
	involvement of other organs and systems *	gastrointestinal, skin, mucosal, respiratory, renal, neurologic	arthritis, brain (chorea), and skin
	high inflammation markers *	CRP, ESR, ferritin, PCT	CRP, ESR
Pathogenesis	genetic predisposition	suspected	yes
	molecular mimicry	suspected	yes
	high levels of pro-inflammatory cytokines	yes	yes
	increased activity of T lymphocytes	yes	yes
Clinical symptoms	mild course of primary infection	yes	yes
	age of onset	4–17 years	5–14 years, rare in adults
	time from exposure to illness	3–6 weeks	2–3 weeks
Treatment	eradication of the pathogen	yes, if confirmed	yes
	anti-inflammatory, corticosteroids	yes	yes
	acetylsalicylic acid	yes, antithrombotic doses	yes, anti-inflammatory doses
	antibiotic therapy	no	primary and secondary prevention
	IVIG	yes	possible but not routine
	biological drugs	anti IL-6, IL-1, TNF	no
	good response to treatment	yes	yes
Course and prognosis	course	no data, probably one course	may be recurrent, *GAS* infection prevention (secondary) required
	prognosis	no data, advised observation, especially of the cardiovascular system	chronic and progressive rheumatic heart disease in 50% patients with carditis
Prevention	vaccination	no data, available for adults	ongoing research

* diagnosis criterion; *SARS-CoV-2*, severe acute respiratory syndrome coronavirus 2; PIMS-TS—paediatric inflammatory multisystem syndrome temporally associated with *SARS-CoV-2*; ARF, acute rheumatic fever; *GAS*, group A streptococcus; ASO, Anti-streptolysin O; CRP, C-reactive protein; ESR, erythrocyte sedimentation rate; PCT, procalcitonin; IL, interleukin; TNF, tumor necrosis factor; IVIG, intravenous immunoglobulin.
